# QRFP-Deficient Mice Are Hypophagic, Lean, Hypoactive and Exhibit Increased Anxiety-Like Behavior

**DOI:** 10.1371/journal.pone.0164716

**Published:** 2016-11-11

**Authors:** Kitaro Okamoto, Miwako Yamasaki, Keizo Takao, Shingo Soya, Monica Iwasaki, Koh Sasaki, Kenta Magoori, Iori Sakakibara, Tsuyoshi Miyakawa, Michihiro Mieda, Masahiko Watanabe, Juro Sakai, Masashi Yanagisawa, Takeshi Sakurai

**Affiliations:** 1 Department of Molecular Neuroscience and Integrative Physiology, Faculty of Medicine, Kanazawa University, Kanazawa, Ishikawa 920–8640, Japan; 2 Department of Anatomy, Graduate School of Medicine, Hokkaido University, Sapporo, Hokkaido 060–8638, Japan; 3 Section of Behavior Patterns, Center for Genetic Analysis of Behavior, National Institute for Physiological Sciences, 38 Nishigonaka Myodaiji, Okazaki, Aichi, 444–8585, Japan; 4 Division of Animal Resources and Development, Life Science Research Center, University of Toyama, Toyama, 930–8555, Japan; 5 Division of Metabolic Medicine, Research Center for Advanced Science and Technology (RCAST), The University of Tokyo, Tokyo 153–8904, Japan; 6 ERATO Yanagisawa Orphan Receptor Project, Japan Science and Technology Agency, Tokyo 135–0064, Japan; 7 Division of Systems Medical Science, Institute for Comprehensive Medical Science, Fujita Health University, Toyoake, 470–1192, Japan; 8 Faculty of Medicine/International Institute for Integrative Sleep Medicine (IIIS), University of Tsukuba, Tsukuba 305–8575, Japan; Kent State University, UNITED STATES

## Abstract

How the hypothalamus transmits hunger information to other brain regions to govern whole brain function to orchestrate feeding behavior has remained largely unknown. Our present study suggests the importance of a recently found lateral hypothalamic neuropeptide, QRFP, in this signaling. *Qrfp*^-/-^ mice were hypophagic and lean, and exhibited increased anxiety-like behavior, and were hypoactive in novel circumstances as compared with wild type littermates. They also showed decreased wakefulness time in the early hours of the dark period. Histological studies suggested that QRFP neurons receive rich innervations from neurons in the arcuate nucleus which is a primary region for sensing the body’s metabolic state by detecting levels of leptin, ghrelin and glucose. These observations suggest that QRFP is an important mediator that acts as a downstream mediator of the arcuate nucleus and regulates feeding behavior, mood, wakefulness and activity.

## Introduction

Since leptin’s discovery, dozens of hypothalamic neuropeptides have been shown to be involved in the regulation of food intake and body weight homeostasis as downstream players in leptin signaling [1,2]. Especially, neuropeptide Y (NPY), agouti-related peptide (AgRP), α-melanocyte stimulating hormone (α-MSH), and cocaine- and amphetamine-regulated transcript (CART) have been extensively studied [2]. These peptides are produced by neurons resident in the hypothalamic arcuate nucleus, which is a primary region for sensing the body’s metabolic state by detecting levels of leptin, ghrelin and glucose. Two types of leptin-responsive neurons in the arcuate nucleus are thought to play crucial roles [1]. Among the arcuate neurons, those producing two of these peptides, pro-opiomelanocortin (POMC), the precursor of α-MSH, and CART (POMC/CART neurons), are thought to inhibit feeding. Conversely, arcuate neurons producing NPY, AgRP and gamma-amino butyric acid (GABA) (NPY/AgRP/GABA neurons) promote feeding. Leptin stimulates POMC/CART neurons, while suppressing NPY/AgRP/GABA neurons to decrease feeding [1,2]. Recently, the parabrachial nucleus (PBN) [3,4] and paraventricular nucleus (PVN) [5] were shown to be innervated by arcuate neurons and play important roles in the regulation of feeding behavior.

Information about amount of body's energy store is thus gathered by the Arc, and then transmitted to second order neurons, including ones localized in the paraventricular nucleus and others in the lateral hypothalamic area (LHA), to evoke hunger sensation and feeding behavior. Since the seminal study by Anand and Brobeck identified the LHA as the“feeding center”, this region has been thought to play an important role in the regulation of feeding behavior [6]. However, the roles and mechanisms of LHA neurons in regulation of feeding behavior have remained unclear. One of the widely accepted current understandings about the role of the LHA in the regulation of feeding is that it provides output from the hypothalamus to control appetite and metabolic rate, by sending information to various regions of the brain [7]. Included in these projections are orexin- and melanin concentrating hormone (MCH)-producing neurons, which are distinct populations of neurons distributed in and around the LHA, and both project diffusely to the entire central nervous system [8,9]. Several studies have suggested that orexin-producing neurons play a role in supporting arousal aspects of feeding behavior [10,11], while MCH-producing neurons are likely to play important roles in the emotional and motivational aspects of feeding [12]. However, how hunger information gathered in the LHA is transmitted to other brain regions to evoke desire for food has remained largely undisclosed.

QRFP, also known as P518 or 26RFa, is a recently identified neuropeptide, selectively produced by neurons located in the LHA and adjacent regions [13–15]. It was initially found as an endogenous ligand of a human G-protein coupled receptor, GPR103, by a bioinformatics approach. Our group purified endogenous peptide ligand for hGPR103 from rat brain by reverse pharmacology as a 43-residue RF-amide polypeptide [15]. We and other groups have reported that central administration of QRFP potently induced food intake in mice [15,16], rats[17–19], and birds [20]. *QRFP* mRNA was upregulated by fasting, and downregulated when mice were fed a high-fat diet (HFD) [15,21]. HFD feeding increased expression of *QRFP* mRNA in the hypothalamus of female rats, and estradiol, which is a potent regulator of feeding behavior was shown to increase *QRFP*[22]. From these observations, we hypothesized that this neuropeptide is involved in the physiological mechanisms of feeding behavior and energy homeostasis.

In this study, we examined the phenotype of *Qrfp*^-/-^ mice, which are markedly hypophagic and lean. These mice also showed increased anxiety-like behavior. We also histologically identified neuronal input of QRFP-producing neurons. These histological and functional findings clearly identified an important, previously unidentified neuronal pathway that is critically involved in the regulation of mood and feeding behavior.

## Materials and Methods

### Animals

**Fig 1:**
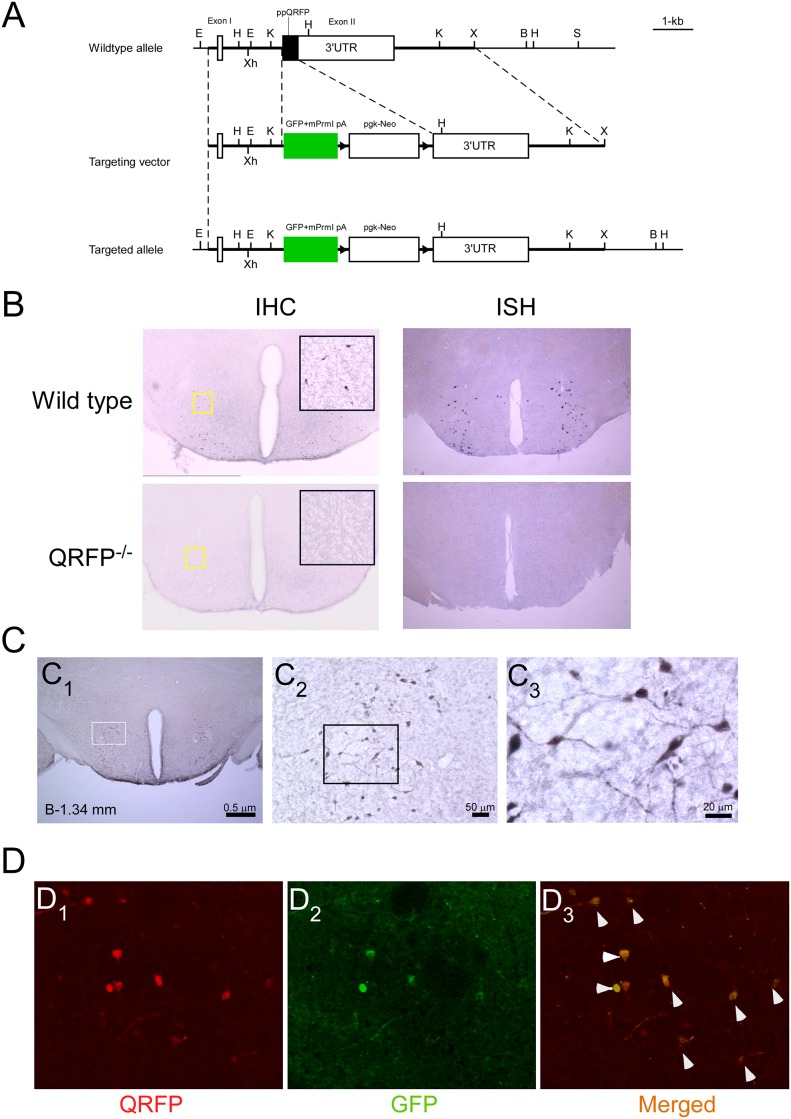
Strategy and characterization of mouse QRFP gene disruption. **A,** Strategy for QRFP disruption. B, BamHI; E, EcoRI; H, HindIII; K, KpnI; S, SalI; X, XbaI; Xh, XhoI. GFP, green fluorescent protein; mPrm1, a part of second exon of the murine protamine-1 gene, which contains an intron and a polyadenylation site **B,** Immunohistochemistry (left panels) and in situ hybridization (right panels) of coronal sections of brains from wild type (upper panels) and *QRFP*^-/-^ mice (lower panels), showing complete depletion of QRFP expression in *QRFP*^-/-^ mice. Insets in left panels show high power views of corresponding yellow rectangular region in each panel. **C,** Distribution of QRFP neurons revealed by immunostaining with anti-GFP antibody in hypothalamic slice of *Qrfp*^-/+^ (GFP knock-in) mouse. C_1_, Coronal brain section of *Qrfp*^-/+-^ mouse, stained with anti-GFP antiserum. C_2_, Higher power view of rectangular region shown in C_1_. C_3_, Higher power view of rectangular region shown in C_2_. **D,** Immunostaining of hypothalamic slice of *Qrfp*^-/+^ brain with anti-GFP and anti-QRFP antibodies revealed that more than 95% of QRFP neurons were labeled with anti-GFP antibody, without any evidence of ectopic expression. D_1_, QRFP-like immunoreactivity. D_2_, Immunofluorescent image of GFP. D_3_, Merged image of D_1_ and D_2._

*Qrfp*^*-/-*^ (eGFP knock-in) mice were generated by homologous recombination in embryonic stem cells of 129SvJ strain and implanted in C57 blastocysts using standard procedures. We constructed the targeting vector by replacing entire conding region of prepro-QRFP sequence in the exon 2 of QRFP gene with GFP sequenc and pgk-Neo cassette (Fig 1A). Since we found GFP fluorescence is effectively expressed in QRFP neurons without any ectopic expression (Fig 1B), we used mice without deleting the pgk-Neo cassette. Genotypes were determined by PCR of mouse tail DNA. PCR primers used were 5’-CAGTCAGCAGCTATCCCTCC-3’ (from-115 to -96base of the QRFP gene from transcription initiation site) and 5’-ACCGTCTTGCCTCCCTAGACG-3’ (from 225 to 246base), and 5’-TCAGCTCGATGCGGTTCAC-3’ (corresponding to the GFP sequence). We detected 361-bp product from wild type allele, and 450-bp product from the targeted allele. Chimeric mice were crossed with C57B/6J females (Jackson Labs). Initially, F1 hybrids from heterozygous x heterozygous mating were generated. They were crossed with C57B/6J mice for more than 10 generations. *Qrfp*^*-/-*^ mice and wild type control littermates were basically obtained by heterozygous x heterozygous. For behavioral tests, we obtained *Qrfp*^*-/-*^ mice and wild type mice by closing homozygous x homozygous to obtain large numbers of mice with the same genotypes and age. Animals were housed at a constant 23°C with a 12 h light/dark cycle (lights off at 20:00), with food and water available ad libitum unless otherwise stated. Mice were housed at three to five per cage. Unless otherwise stated, all tests were conducted with naive cohorts of mice. All experimental procedures were reviewed and approved by the Kanazawa University Institutional Animal Care and conducted in accordance with NIH guidelines.

### Body weight and food intake

For measurements of body weight and food intake, were housed singly. Food intake and body weight were measured every week. Composition of high fat diet used in this study is protein 25.5, fat 32.0, and carbohydrate 29.4 (%) (507.6 kcal/100 g) (HFD32, Clea, Japan) (S1 Table).

### Leptin measurement

Serum leptin levels were measured by ELISA assay using the mouse/rat leptin assay system (Morinaga Institute of Biological Science, Japan).

### Antibodies

We used the following primary antibodies: guinea pig anti-QRFP antibody, which was raised against full-length mouse QRFP-43 sequence (QRFP 81–123 aa (NM_183424)), which detects both QRFP-26 and QRFP-43, rabbit anti-GFP [23], goat anti-AgRP (R&D Systems), and sheep anti-αMSH (Millipore Bioscience Research Reagents). Rabbit NPY antibody was produced against glutathione S-transferase (GST) fusion proteins containing 29–64 amino acid residues of mouse NPY (NM_023456), and affinity-purified[24]. Specificity of this antibody was verified using brain slices from *NPY*^-/-^ mice (S1 Fig).

### Immunohistochemistry

Mice were fixed transcardially with 4% paraformaldehyde in 0.1 M sodium phosphate buffer (pH 7.2, PB) for 10 min. Coronal sections of fixed brains (50-μm thickness) were prepared and subjected to free-floating immunohistochemical staining. All immunohistochemical incubations were performed at room temperature. For immunofluorescent staining, sections were incubated successively with 10% normal donkey serum for 20 min, a mixture of primary antibodies overnight (1 μg/ml for each) at 4°C, and a mixture of Alexa 488-, Cy3-, or Cy5-labeled species-specific secondary antibodies for 2 h at a dilution of 1:200 (Invitrogen; Jackson ImmunoResearch). Images were taken with a confocal laser scanning microscope FV1000 (Olympus) or a fluorescent microscope BZ-9000 (Keyence).

For pre-embedding double-labeling immunoelectron microscopy, microslicer sections (50-μm thickness) were incubated with a mixture of primary antibodies (1 μg/ml each), followed by incubation with biotinylated donkey anti-sheep or goat IgG (Jackson ImmunoResearch) for 2 hr, and streptavidin-peroxidase complex for 1 hr (Nichirei). After immunoperoxidase reaction, sections were subjected to incubation with a secondary antibody linked to 1.4-nm gold particles (Nanogold; Nanoprobes) overnight. After intensive washing in 0.004% saponin/PBS, sections were treated with silver enhancement solution (R-Gent silver enhancement kit; Aurion) for 90 min. Sections were further fixed with 1% osmium tetroxide for 15 min, stained with 2% uranyl acetate for 30 min, dehydrated, and embedded in Epon 812. Electron micrographs were taken with a JEM1400 (JEOL).

For double-labeling with immunogold postembedding, microslicer sections (250 μm in thickness) were first subjected to immunoperoxidase for GFP labeling; sections were incubated successively with 10% normal donkey serum for 20 min, and goat anti-GFP (1 μg/ml) overnight at room temperature. Sections were further incubated with biotinylated donkey anti-goat IgG (Jackson ImmunoResearch) for 2 hr, and streptavidin-peroxidase complex for 1 hr (Nichirei). Immunoreaction was visualized with DAB. Sections were cryoprotected with 30% glycerol in PB, and frozen rapidly with liquid propane in an EM CPC unit (Leica Microsystems). Frozen sections were immersed in 0.5% uranyl acetate in methanol at −90°C in an AFS freeze-substitution unit (Leica Microsystems), infiltrated at −45°C with Lowicryl HM-20 resin (Chemische Werke Lowi), and polymerized with UV light. Ultrathin sections on nickel grids were etched with saturated sodium ethanolate solution for 1–5 s, and treated successively with blocking solution [2% normal goat serum (Nichirei) in 0.03% Triton X-100 in Tris-buffered saline (TBST; pH 7.4)] for 20 min, rabbit anti-NPY (20 μg/ml) diluted with the same blocking solution overnight, and colloidal gold (20 nm)-conjugated rabbit anti-goat IgG (1:100, British BioCell International) for 2 hr. After washing with TBST, grids were fixed with 2% glutaraldehyde in PB for 15 min and 1% OsO_4_ for 20 min, and stained with 2% uranyl acetate for 10 min and Reynold’s lead citrate solution for 1 min. Electron micrographs were taken with a JEM1400 (JEOL).

### In situ hybridization

In situ hybridization was performed as previously described (Mieda et al., 2006). Digoxigenin (DIG)-labeled riboprobes for QRFP were synthesized by RT-PCR from a 0.60-kb fragment of mouse cDNA encoding prepro-QRFP from mouse brain RNA and subcloned into pCRII vector (Invitrogen). The following primers were used to amplify cDNA fragments: prepro-QRFP, 5'-CCTCCCACAGGGAGCACACCG-3' and 5'-CCTGTTGTATCCACGGCCCCA-3'. The DIG-labeled probes were detected by anti-DIG (1/1000) antibodies conjugated with alkaline phosphatase (Roche Diagnostics, Basel, Switzerland). Alkaline phosphatase activity was detected with NBT/BCIP (Roche Diagnostics).

### Indirect calorimetry

Energy expenditure was measured as described previously [15]. In brief, oxygen consumption was measured with an O_2_/CO_2_ metabolism-measuring system (model MK-5000, Muromachikikai). Each mouse was placed in a sealed chamber (560-ml volume) with an air flow of 0.60 liters/min for 22 h at 23 C. Air was taken every 3 min, and the consumed oxygen concentration was converted to milliliters per minute by multiplying it by the flow. Respiratory quotient, the ratio of CO_2_ production to oxygen consumption was also measured.

### Computed tomography

Images were obtained using a computed tomographic scanner for mice (Shimazu, Japan), and analyzed with VGStudioMAX software.

### Behavior analyses

#### Animals and experimental design

All behavioral tests were carried out in male mice that were at least 9 weeks old at the start of testing. Mice were group-housed (2–4 mice per cage) in a room with a 12-h light/dark cycle (lights on at 07:00 hours) with access to food and water ad libitum. Room temperature was kept at 23±2°C. Behavioral testing was performed in the light period. Elevated plus maze test, openfield test, and light-dark transition test were performed at 10:00–16:00, 08:00–14:00, and 10:00–15:00, respectively. After the tests, all apparatuses were cleaned with diluted sodium hypochlorite solution to prevent a bias due to olfactory cues. All behavioral tests were separated from each other by at least one day. All behavioral testing procedures were approved by the Animal Research Committee, National Institute for Physiological Sciences.

#### Locomotor activity monitoring in home cage

A system that automatically analyzes the locomotor activity of mice in their home cage was used. The system contained a home cage (29×18×12 cm) and a filtered cage top, separated by a 13 cm-high metal stand containing an infrared video camera, which was attached to the top of the stand. Each mouse was individually housed in a home cage, and its locomotor activity was monitored for 1 week. Outputs from the video cameras were fed into a computer. Images from each cage were captured at a rate of one frame per second, and distance travelled was measured automatically using ImageHA software (see “Image analysis”).

#### Elevated plus maze test

The elevated plus maze apparatus consisted of two open arms (25×5 cm) and two enclosed arms of the same size, with transparent walls 15 cm high (Komada et al, 2008). The arms and central square were made of white plastic plates and were elevated 55 cm above the floor. To minimize the likelihood of animals falling from the apparatus, 3-mm-high plexiglass ledges were provided for the open arms. Arms of the same type were arranged on opposite sides. Each mouse was placed in the central square of the maze (5 × 5 cm) facing one of the closed arms. Behavior was recorded during a 10 min test period. The lighting value was 100 lx. The number of entries into and the time spent in open and enclosed arms were recorded. For data analysis, the following four measures were employed: the percentage of entries into open arms, the time stayed in the open arms (s), the total number of entries, and the total distance traveled (cm). To specify the locations of the mice, the center of balance was used (i.e., "entry" indicates that the center of the mass of the mouse enters the other arm). Data acquisition and analysis were performed automatically, using an ImageJ-based original program (ImageEP: see “Image analysis”).

#### Open field test

Locomotor activity was measured using an open field test. Each mouse was placed in one corner of the open field apparatus (40×40×30 cm; Accuscan Instruments, Columbus, OH). Total distance traveled (in cm), vertical activity (rearing measured by counting the number of photobeam interruptions), time spent in the center, the beam-break counts for stereotyped behaviors. The lighting value was 100 lx. Data were collected for 120 min.

#### Tail suspension test

Each mouse was suspended 30 cm above the floor by the tail in a white plastic chamber (31×41×41 cm) (O’Hara & Co., Tokyo, Japan). The behavior was recorded for 10 min. Images were captured at one frame per second. For each pair of successive frames, the area (pixels) within which the mouse moved was measured. When the area was below a certain threshold, the mouse was judged to be "immobile". When the area equaled or exceeded the threshold, the mouse was considered to be "moving". The optimal threshold was determined by adjusting it to the degree of immobility measured by human observation. Immobility lasting for less than 2 s was not included in the analysis.

Image analysis: The applications used for the behavioral studies (Image HA, Image EP, and Image TS) were based on the public domain ImageJ program (http://rsb.info.nih.gov/ij/) and were modified for each test by the authors (available through O'Hara & Co., Tokyo, Japan). ImageEP is freely available at the following URL: http://www.mouse-phenotype.org/software.html.

### Sleep recordings

An electrode for EEG and EMG recording was implanted in the skull of each mouse as described previously [25]. The three arms of the electrode for EEG recording were placed approximately 2 mm anterior and 2 mm to the right, 2 mm posterior and 2 mm to the right, and 2 mm posterior and 2 mm to the left of the bregma. Stainless steel wires for EMG recording were sutured to the neck muscles of each mouse bilaterally, and each electrode was glued solidly to the skull. After the recovery period, animals were moved to a recording cage placed in an electrically shielded and sound attenuated room. A cable for signal output was connected to the implanted electrode, and animals were allowed to move freely. Signals were amplified through an amplifier (AB-611J, Nihon Koden, Tokyo) and digitally recorded on a computer using EEG/EMG recording software (Vital recorder, Kissei Comtec). Animals were allowed at least 7 days to adapt to the recording conditions prior to any EEG/EMG recording session.

### Statistical analysis

Data in graphs are expressed as mean ± SEM. Groups were compared using two-tailed t-test, two-way ANOVA, or two-way repeated measures ANOVA with Bonferroni correction as a post hoc analysis. Probability levels <0.05 were considered statistically significant. Statistical testing was performed using StatView (SAS Institute, Cary, NC).

## Results

### QRFP-deficient mice are lean and hypophagic

To examine the physiological role of QRFP, we generated QRFP gene-deficient (*Qrfp*^-/-^) mice by replacing the entire prepro-QRFP sequence with the enhanced green fluorescent protein (eGFP) gene, so that we could easily identify QRFP-producing cells in the knock-in mice (Fig 1A). We raised specific anti-serum to against full-length synthetic QRFP-43 peptide and used it for immunostaining, and again obtained a similar distribution of positively stained neurons in wild type slices (Fig 1B). We could not found any significant staining above the background level in *Qrfp*^-/-^ slices. We previously showed that QRFP-producing neurons were exclusively localized in the hypothalamic regions, including the LHA, perifornical region, and tuber cinereum [15]. We confirmed this distribution by immunostaining with anti-GFP antibody using brain slices from *Qrfp*^-/+^ (heterozygous GFP knock-in) mice (Fig 1C). Immunostaining for QRFP in *Qrfp*^-/+^ mice showed that GFP-positive neurons completely overlapped with QRFP-positive cells (Fig 1D).

**Fig 2:**
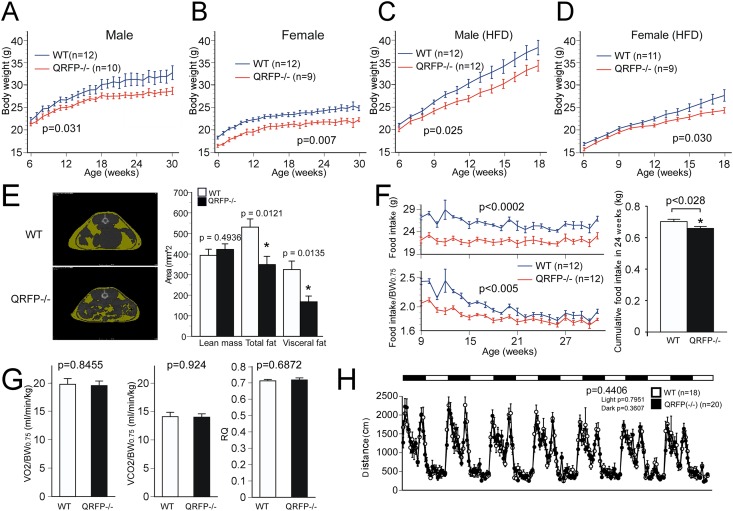
*Qrfp*^-/-^ mice are lean and hypophagic. **A, B,** Body weights of male (**A**) and female (**B**) *Qrfp*^-/-^ mice (red lines) under ad lib feeding of normal chow compared with wild type littermates (blue lines) plotted over time. **C, D,** Body weights of male (**C**) and female (**D**) *Qrfp*^-/-^ mice (red lines) under feeding a high-fat diet compared with wild type littermates (blue lines). High-fat diet contained 56.7% fat (High Fat Diet 32, CLEA Japan Inc., Tokyo, Japan). **E,** Left panel, Representative CT images of age-matched (15 weeks) wild type (upper panel) and *Qrfp*^-/-^ mice. Fat mass is shown in yellow. Right panel, Lean and fat mass of *Qrfp*^-/-^ mice compared with wild type littermates (n = 5). *, p<0.05. **F,** Weekly food intake of *Qrfp*^-/-^ mice (red lines) under ad lib feeding of normal chow compared with wild type littermates (blue lines) plotted over time. Left upper panel, Total weekly food intake over time. Left lower panel, Weekly food intake normalized by 0.75 power of body weight. Right panel, Cumulative food intake for 24 weeks (9 to 33 weeks of age) of *Qrfp*^-/-^ mice (closed bars) under ad lib feeding of normal chow compared with wild type littermates (open bars). **G,** Basal energy expenditure of *Qrfp*^-/-^ mice (closed bars, n = 6) is comparable to that of wild type mice (open bars, n = 6). Left panel, V_O2_ normalized by 0.75 power of body weight. Middle panel, V_CO2_ normalized by 0.75 power of body weight. Right panel, Respiratory quotient (RQ) of wild type mice vs *Qrfp*^-/-^ mice. **H,** Daily locomotor activity of *Qrfp*^-/-^ mice, which was comparable to that of wild type mice.

We found that the body weight of *Qrfp*^-/-^ mice was significantly lower than that of both male and female wild type controls under ad lib feeding (Fig 2A and 2B). Body weight of *QRFP*^-/-^ mice was lighter as compared with wild type mice, by 8.3% for male and (WT, n = 12; KO, n = 10, F_1,20_ = 5.591, P = 0.0423) 9.6% for female mice (WT, n = 12; KO, n = 9, F_1,19_ = 9.728, p = 0.0206) at 18 weeks of age. The body weight of *Qrfp*^-/-^ mice under high-fat chow feeding was also significantly lower (Fig 2C and 2D), by 10.6% for male (WT, n = 12; KO, n = 12, F_1,22_ = 5.475, p = 0.0288) and 11.9% for female mice (WT, n = 11; KO, n = 9, F_1,19_ = 4.943, p = 0.0392) at 18 weeks of age compared to wild type controls fed the same diet. Computed tomography showed that *Qrfp*^-/-^ mice on a normal diet had lower visceral and total fat mass at 30 weeks of age (Fig 2E) (n = 5, total fat, F_1,8_ = 10.415, p = 0.0121; subctaneous fat, F_1,8_ = 0.627, p = 0.4512; visceral fat, F_1,8_ = 9.954, p = 0.0135). Lean mass was comparable to that of controls (n = 5, F_1,8_ = 0.515, p = 0.4936), suggesting they did not suffer from growth abnormality. Consistently, serum leptin level of *Qrfp*^-/-^ mice at 30 weeks of age was also significantly lower than that of wild type mice (9.98±1.46 and 3.10±0.71 ng/ml for wild type and *Qrfp*^-/-^ mice at 30 weeks of age, respectively, n = 4, F_1,6_ = 17.832, p = 0.0055).

Male *Qrfp*^-/-^ mice ate on average 6% less as compared to control mice on a normal chow diet from 9 to 33 weeks of age (n = 12, F_1,22_ = 13.622, p = 0.005) (Fig 2F). Metabolic rate measured as VO_2_ of *Qrfp*^-/-^ mice was not significantly different from that of wild type controls (n = 6, VO_2_, F_1,10_ = 0.04, p = 0.8455;VCO_2_, F_1,10_ = 0.009, p = 0.9427; RQ, F_1,10_ = 0.172, p = 0.6870) (Fig 2G). Their daily spontaneous locomotor activity was not significantly different from that of control mice, either (WT, n = 18; KO, n = 20) (Fig 2H). From these observations, we concluded that *Qrfp*^-/-^ mice are lean due to hypophagia, with energy expenditure remaining normal.

### QRFP neurons receive innervation from arcuate NPY/AgRP neurons

**Fig 3:**
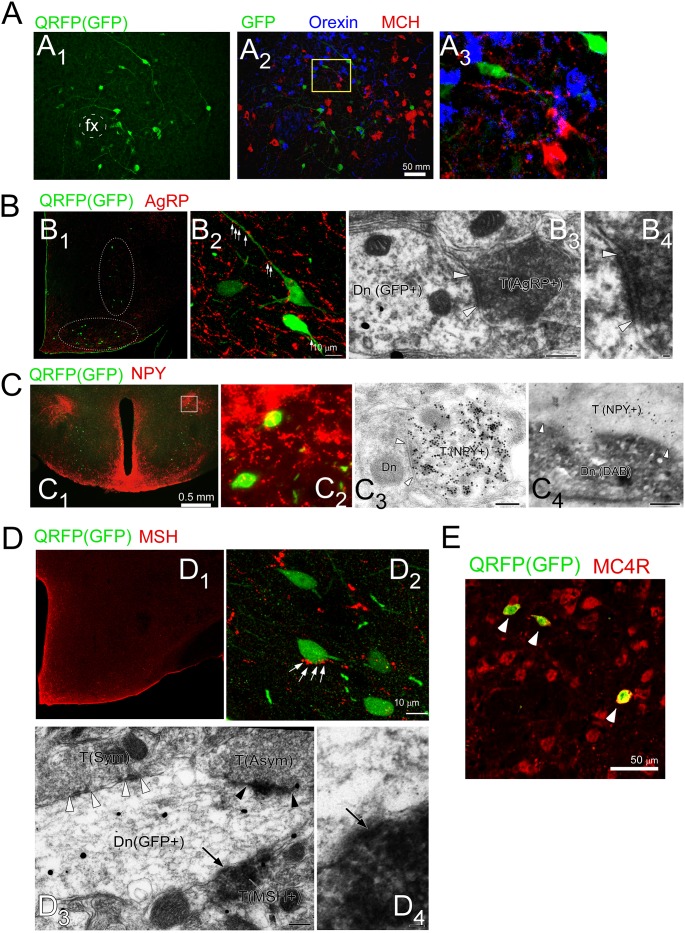
QRFP-producing neurons are distributed in and around the LHA and innervated by fibers sent by arcuate neurons. **A,** Triple immunofluorescent image of QRFP neurons (GFP, green), orexin-ir neurons (blue) and MCH-ir neurons (red) in hypothalamic slice of *QRFP*^+/-^ mouse. A_1_, QRFP neurons (GFP, green) of perifornical region of *QRFP*^+/-^ mouse. Fx, fornix. A_2_, Triple immunofluorescent image of QRFP neurons (GFP, green), orexin-ir neurons (blue) and MCH-ir neurons (red) in same section as in A_1_. A_3_, High power view of yellow rectangular region in A_2_. **B,** QRFP neurons receive innervations from AgRP-immunoreactive (ir) fibers. Double immunofluorescence for QRFP neurons (GFP, green) and AgRP-ir fibers (red) showing dense innervation by AgRP neurons in the hypothalamic area. B_1-2_, Low power (B_1_) and high power (B_2_) views of double-labeling immunofluorescent image of GFP (green) and AgRP immunoreactivity in hypothalamic area. B_3_, double-labeled pre-embedding immunoelectron microscopic image of AgRP (diffuse precipitates) and GFP (metal particles) showing a symmetrical synapse between a QRFP neuron and an AgRP neuron. B_4_, High power view of immunoelectron microscopic image showing a synapse between a dendrite of a QRFP neuron and a terminal of an AgRP fiber. White arrowheads indicate the edge of postsynaptic density of symmetrical synapse. **C,** Innervation from NPY neurons to QRFP neurons. C_1_, Double immunofluorescent images of QRFP neurons (GFP, green) and NPY-ir fibers (red) in a hypothalamic slice of *QRFP*^-/+^ mouse. C_2_, High power view of region shown in yellow rectangle. C_3-4_, Low power (C_3_) and high power (C_4_) views of postembedding immunoelectron microscopy for NPY (immunogold) showing a symmetrical synapse between an NPY-ir terminal and a GFP-positive neuron. White arrowheads indicate the edge of postsynaptic density. **D,** Close interaction between QRFP neurons and αMSH-ir fibers. Presence of QRFP neurons (GFP, green) and αMSH-ir fibers (red) in a hypothalamic slice of *QRFP*^-/+^ mouse, showing QRFP neurons are closely apposed to αMSH-ir fibers. D_1_, Low power view of a hypothalamic slice stained with anti-αMSH antibody. D_2_, High power view of hypothalamic slice stained with anti-αMSH (red) and anti-GFP antibodies. D_3-4_, Low power (D_3_) and high power (D_3_) views of double-labeling pre-embedding immunoelectron microscopic image showing αMSH (diffuse precipitates) and GFP (metal particles). Black and white arrowheads indicate the edge of postsynaptic density of asymmetrical and symmetrical synapse, respectively. **E,** Double labeling immunofluorescent image showing expression of MC4 melanocortin receptor (MC4R, red) in QRFP neurons (GFP, green) in a coronal section of an *Qrfp*^-/+^ slice.

Triple immunofluorescent labeling study showed that these GFP-positive neurons comprise a distinct population from orexin neurons and MCH neurons in the LHA, suggesting that QRFP neurons do not express orexin or MCH and that they play distinct roles in the LHA (Fig 3A).

Double immunofluorescent staining of hypothalamic slices from *Qrfp*^-/-^ mice with anti-AgRP and anti-GFP antibodies showed that AgRP-immunoreactive (AgRP-ir) punctate and varicose fibers were distributed mainly in the paraventricular nucleus and LHA regions (Fig 3B). The area of distribution of AgRP-ir fibers in the hypothalamus overlapped with that of QRFP-positive neurons. AgRP-ir varicosities were distributed with no particular gradient toward QRFP neurons; however, all QRFP-positive neurons had close associations with AgRP-ir varicosities (25 out of 25 cells; Fig 3B). Double-labeled immunoelectron microscopy further revealed that AgRP-positive terminals formed symmetrical synapses on GFP-positive dendrites in *Qrfp*^-/-^ mice (Fig 3B_3_ and 3B_4_). We also observed similar synaptic contact between NPY-ir varicosities and QRFP neurons (Fig 3C), and double-labeling immunoelectron microscopy revealed that NPY-ir terminals and QRFP neurons were directly apposed to each other (Fig 3C_3_ and 3C_4_). Double immunofluorescent staining for αMSH and GFP showed that αMSH-ir fibers were also distributed in the hypothalamus, especially in subregions where QRFP neurons are located (Fig 3D), although αMSH-positive fibers were far sparser than AgRP-ir counterparts. They showed no clear accumulation toward QRFP neurons, and only a small population of QRFP neurons were in close apposition with αMSH-positive terminals (6 out of 32 cells; Fig 3D_2_). Further analysis by double-labeling immunoelectron microscopy revealed that even where αMSH-ir terminals and QRFP neurons were directly apposed to each other, they did not show discernible postsynaptic specialization (Fig 3D_3_ and 3D_4_). However, these observations do not exclude an interaction between αMSH and QRFP neurons, and suggest the possibility that αMSH-ir terminals might release their peptide with a mode of volume transmission. Supporting this hypothesis, we found that QRFP neurons express MC4 melanocortin receptors (MC4R) by double labeling immunostaining of *Qrfp*^*-/+*^ slices with anti-GFP and anti-MC4R antisera (Fig 3E).

These histological observations identified a close connection between arcuate neurons, especially NPY/AgRP neurons and QRFP neurons. These observations raise the possibility that QRFP neurons are one of the downstream effectors of NPY/AgRP neurons, which are involved in sensing peripheral metabolic states to regulate feeding behavior.

### QRFP is involved in mood, emotion and wakefulness

**Fig 4:**
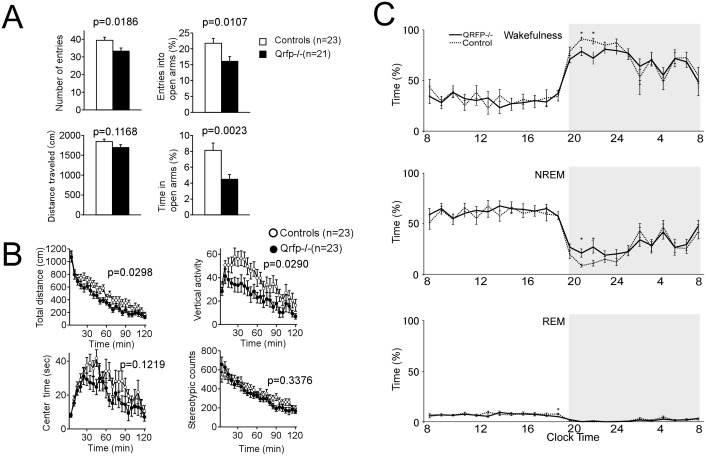
*Qrfp*^-/-^ mice exhibited increased anxiety-like behavior and decreased wakefulness in the early hours of the dark period. **A,** Elevated plus maze test. *Qrfp*^-/-^ mice showed fewer entries into the open arms, and spent a shorter time in the open arms. **B,** Open field test. *Qrfp*^-/-^ mice showed decreased spontaneous horizontal and vertical activities. Stereotypic behavior and time spent in the central area were not significantly different between *Qrfp*^-/-^ mice and wild type mice. **C,** Decreased wakefulness time of *Qrfp*^-/-^ mice in early hours of dark period. Hourly amounts of each sleep state (mean ± SE) are plotted over 24 h for control littermates (*n* = 10) and *Qrfp*^-/-^ mice (*n* = 10). Data for the dark and light phases are displayed on light gray and white backgrounds, respectively. **Upper,** Wakefulness time. **Middle,** NREM sleep time. **Lower,** REM sleep time.

Most substances that affect food intake show effects on mood and motivation simultaneously, and eating disorders are sometimes accompanied by mood or behavioral disorders [26]. These observations suggest that QRFP might also be involved in mood and emotion. To address this possibility, we conducted a battery of behavioral experiments to further elucidate the characteristics of the knockout mice. Behavioral tests revealed that *Qrfp*^-/-^ mice showed increased anxiety-like behavior. The shorter percentage of time spent in open arms in the elevated-plus maze test suggested increased basal anxiety in mice (WT, n = 23; KO, n = 21, Time on open arms, F_1,42_ = 10.531, p = 0.0023;Number of entries, F_1,42_ = 5.997, p = 0.0186; Entries into open arms, F_1,42_ = 7.139, p = 0.0107; Distance traveled, F_1,42_ = 2.564, p = 0.1168) (Fig 4A). Open field test revealed that the mutants also exhibited lower locomotor activity compared with controls when they were put into the open field arena, which was a novel environment for mice (WT, KO, n = 23, Total distance, F_1,44_ = 5.042, p = 0.0298;Vertical activity, F_1,44_ = 5.095, p = 0.0290; Center time, F_1,44_ = 2.488, p = 0.1219; Stereotypic counts, F_1,44_ = 0.940, p = 0.3376) (Fig 4B). *Qrfp*^-/-^ mice also showed mild abnormality in the tail suspension test, suggesting the possibility that these mice are depressive or hypoactive (data not shown). These observations suggest that QRFP is involved in mood and emotion. The LHA is also critically implicated in the regulation of sleep/wakefulness states. Especially, orexin-producing neurons in the LHA play a highly important role in the maintenance of wakefulness [11], while MCH-containing cells in the region are implicated in the regulation of REM sleep [27–29]. To address the possibility that QRFP is also involved in the regulation of sleep/wakefulness states, basal sleep/wakefulness states of *Qrfp*^-/-^ mice were examined by simultaneous EEG/EMG recording and scoring. Although the overall amounts of wakefulness, non-rapid eye movement (NREM) sleep, and REM sleep in 12-hour light and dark periods were not significantly different between *Qrfp*^-/-^ mice and controls (Wake: *F*_(1, 9)_ = 3.671, *p* = 0.0876, NREM: *F*_(1, 9)_ = 4.404, *p* = 0.0653, REM: *F*_(1, 9)_ = 5.085, *p* = 0.0506) However, hourly analysis in first 6 h at the dark period showed that *Qrfp*^-/-^ mice exhibited decreased awake time (*F*_(1, 9)_ = 10.16, *p* = 0.0110) especially at the point of 3 h and increased non-REM sleep time (*F*_(1, 9)_ = 9.426, *p* = 0.0134) especially at the point of 2 and 3 h after the start of the dark period as compared with the controls (*n* = 6) (Fig 4C). This decrease in wakefulness might be related to decreased motivation for feeding behavior, because proper vigilance levels are needed for feeding behavior.

## Discussion

The neuronal mechanisms of feeding behavior involve many neuropeptides in the hypothalamus[2]. QRFP was recently identified as a hypothalamic peptide that increased food intake[13–15]. This action is accompanied by increase in activity and cardiovascular functions in mice[15]. This suggested that, similar to orexin, this peptide might increase food intake and energy expenditure simultaneously[30]. Therefore, effects of chronic blockade of QRFP signaling might have complex impact on animals’ body weight homeostasis. Recently, a GPR103 antagonist was shown to acutely decrease food intake in male mice[31]. But long-term effects of QRFP blockade on body weight or other phenotypes have remained unknown. To understand the effect of chronic deficiency of QRFP signaling, we examined here whether genetic deletion of QRFP gene in mice can affect animals’ food intake and body weights.

In this study, we showed that *Qrfp*^-/-^ mice exhibit decreased body weight due to hypophagia under both normal and high-fat-fed conditions (Fig 2). QRFP-deficient mice exhibited a normal metabolic rate and daily locomotor activity (Fig 2G and 2H). Although many neuropeptides have been shown to be involved in the regulation of food intake, cases in which single disruption of a particular neuropeptide gene results in hypophagia and lower body weight are very rare. Other than QRFP, only disruption of MCH has been shown to result in hypophagia and low body weight due to single neuropeptide disruption [32]. These observations suggest that QRFP is one of the important factors in the regulation of feeding behavior.

We also histologically examined input system of QRFP producing neurons to obtain insights into the understanding of the regulatory mechanism of QRFP neurons. We found that QRFP neurons are distributed in the basolateral hypothalamic regions, including the tuber cinereum and lateral hypothalamic area, constituting distinct neuronal population from neurons that produce orexin or MCH. It was reported that the expression of QRFP is detected in the arcuate nucleus and ventrolmedial hypothalamus (VMH) in rats [18,22]. However, we did not detect QRFP mRNA or peptide in these regions in this study. This is consistent with our previous data[15]. We also did not observe GFP-positive neurons in those areas in GFP knock-in mouse (Fig 3). This discrepancy might stem from differences in sensitivities of method used to detect the expression (RT-PCR vs ISH), and species difference.

QRFP neurons receive innervations from NPY/AgRP neurons and POMC neurons in the arcuate nucleus. This is consistent with previous studies by other groups, which showed that QRFP is associated with NPY and AgRP and MC3/4R[33,34]. These previous studies support the idea that QRFP alters feeding behavior by interacting with these systems. We previously identified two QRFP receptors, GPR103A and GPR103B, in rodents, and found that both receptors are expressed in regions implicated in the regulation of feeding behavior and mood [15]. For example, GPR103A is found in the ventromedial hypothalamus and nucleus of the solitary tract, while GPR103B is expressed in the PVN, nucleus of the accumbens, ventral tegmental area and periaqueductal gray. These regions are implicated in feeding behavior as well as mood regulation. These observations suggest that QRFP exerts its activity through these regions, and this factor is likely to be involved in emotional aspects of feeding. In fact, we also found that QRFP-deficient mice showed an anxiety-like phenotype (Fig 4). Factorial analysis of behavior in anxiety-related experiments in animals has shown that different tests reflect different underlying factors [35]. Therefore, the fact that *Qrfp*^−/−^ mice showed such a specific phenotype in the elevated plus maze test and not in open filed test might reflect the fact that these tests measure different dimensions of anxiety-related behaviors.

The LHA contains orexin neurons and MCH neurons, both of which are implicated in the regulation of sleep/wakefulness states. We also found that *Qrfp*^-/-^ mice showed shorter wakefulness time especially early time of the dark period, suggesting that QRFP also plays an important role in arousal regulation. Decreased motivation toward feeding might explain the decreased wakefulness time in *Qrfp*^-/-^ mice.

QRFP peptides are also known to be expressed in peripheral organs. QRFP and GPR103 were shown to be expressed in human adrenal cortex, and QRFP induced aldosterone and cortisol secretion in culture cells[36]. QRFP and GPR103b were also shown to be expressed in adipocytes in mice and suggested to be involved in the regulation of adipogenesis[37]. Moreover, prepro-QRFP mRNA and GPR103a mRNA were shown to be expressed in L6 myotube cells in culture, and QRFP-26 was shown to increase insulin's ability to induce glycogen synthesis and 2-deoxyglucose uptake in these cells[38]. Since our present mouse model is conventional QRFP knockout, these peripheral actions metabolic effects of QRFP might also have a contribution to metabolic phenotype of these mice. This should be examined in future studies.

In summary, we showed that QRFP plays an important role in evoking feeding behavior by transmitting signals from the arcuate nucleus. This pathway might be involved in motivational, arousal and emotional aspects of feeding behavior. We identified a novel and important neuronal pathway involving QRFP to induce feeding that links neurons in the LHA and other brain regions. Future studies using optogenetic/pharmacogenetic approaches might further reveal the mechanisms by which QRFP promotes feeding. Our study also provides a novel target for therapeutic approaches toward not only obesity but also for related metabolic abnormalities and mood disorders.

## Supporting Information

S1 Fig Distribution of neuropeptide Y (NPY)-immunoreactive fibers in brain. Left panel, immunostaining of NPY-immunoreactive fibers in wild type mouse. Right panel, immunostaining of the same condition as the left panel using a brain slice of *Npy*^*-/-*^ mouse.
(TIF)

S1 Table Composition of high fat diet used in this study. (XLSX)
