# Supplementary figures and images for: QRFP-Deficient Mice Are Hypophagic, Lean, Hypoactive and Exhibit Increased Anxiety-Like Behavior
Source: PLoS One. 2016 Nov 11;11(11):e0164716. doi: 10.1371/journal.pone.0164716 (PMC5105951; doi:10.1371/journal.pone.0164716)

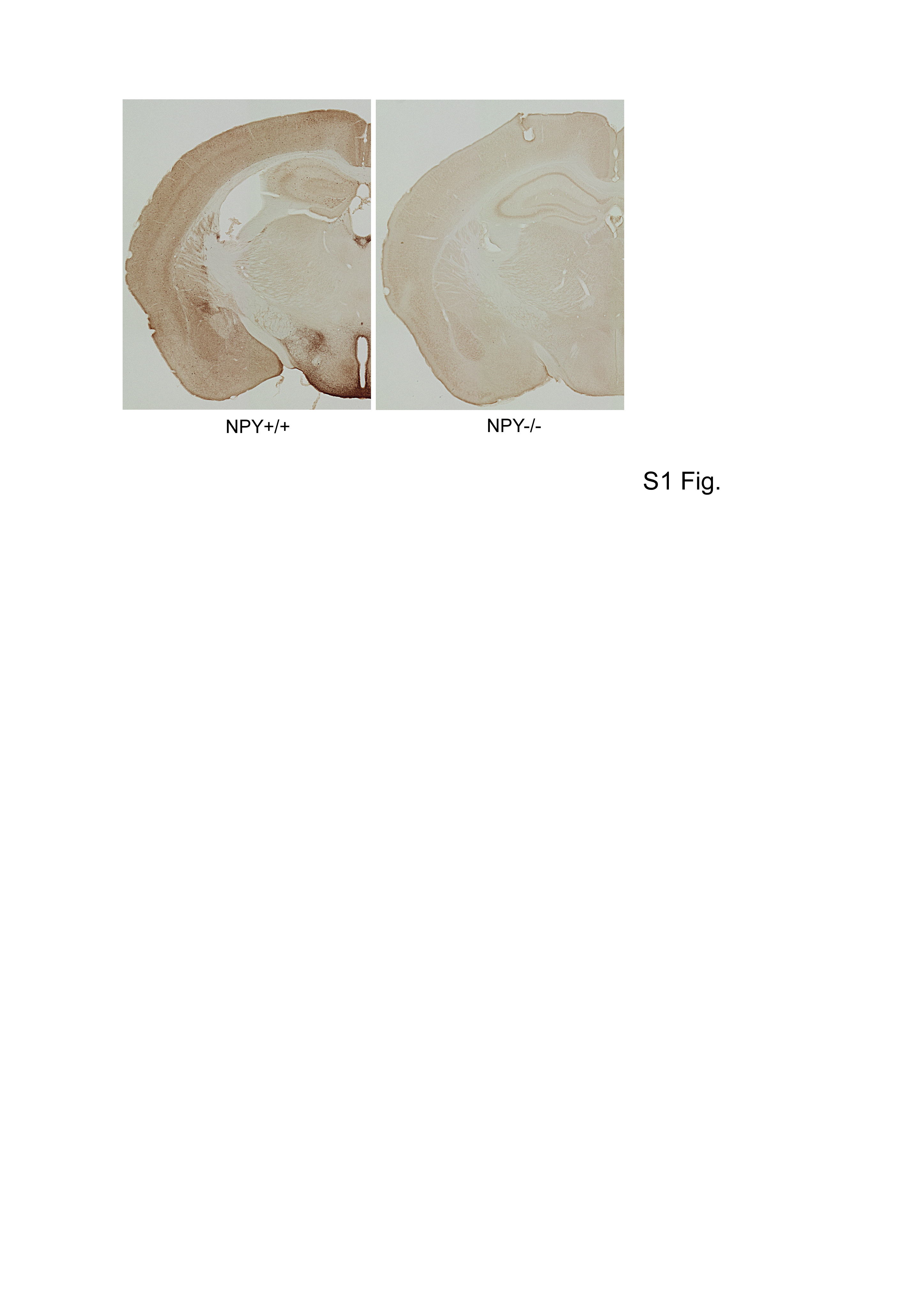

Supplement: S1 Fig — Left panel, immunostaining of NPY-immunoreactive fibers in wild type mouse. Right panel, immunostaining of the same condition as the left panel using a brain slice of Npy-/- mouse. (TIF) [file pone.0164716.s001.tif]
